# Bactericidal ZnO glass-filled thermoplastic polyurethane and polydimethyl siloxane composites to inhibit biofilm-associated infections

**DOI:** 10.1038/s41598-019-39324-w

**Published:** 2019-02-26

**Authors:** Belén Cabal, David Sevillano, Elisa Fernández-García, Luis Alou, Marta Suárez, Natalia González, José S. Moya, Ramón Torrecillas

**Affiliations:** 1 Nanomaterials and Nanotechnology Research Center (CINN-CSIC), Universidad de Oviedo (UO), Principado de Asturias, Avda de la Vega 4-6, 33940 El Entrego, Spain; 2 Nanoker Research, Pol. Ind. Olloniego, Parcela 22A, Nave 5, 33660 Oviedo, Spain; 3 Microbiology Unit, Medicine Department, School of Medicine, Universidad Complutense, Avda. Complutense s/n, 28040 Madrid, Spain

## Abstract

This study investigates a novel approach to controlling biofilms of the most frequent pathogens implicated in the etiology of biomaterials-associated infections. New bactericidal filler based on a non-toxic glass, belonging to B_2_O_3_-SiO_2_-Al_2_O_3_-Na_2_O-ZnO system, was used to formulate composites of the most widely used polymers in biomedical applications [i.e. thermoplastic polyurethane (TPU) and polydimethyl siloxane (PDMS)], with varying percentage by weight of the bactericidal glass (5, 15, 25, 35, 50%). Glass-filled polymer composites show dramatically restricted bacterial colonisation and biofilm formation. They exhibit time- and dose-dependent killing, with maximal action at 5 days. The highest activity was found against *S*.*epidermidis* biofilm (99% of reduction), one of the most common cause of nosocomial infections. The tensile properties of the obtained glass-filled composites are comparable with the literature data concerning polymeric biomaterials for medical implants and devices. In addition, all the materials presented in this research, revealed an excellent biocompatibility. This was disclosed by cell viability values above 70%, none alteration on erythrocyte membrane or cell functionality in contact with materials (haemolytic index 0–2%), and absence of interferences in blood coagulation (intrinsic, extrinsic and final pathways).

## Introduction

Despite recent advances and the success of several initiatives to improve education and hygiene practices, healthcare-associated infections (HAIs) pose continued diagnostic and therapeutic challenges to even well-trained and experienced clinicians. The European Center for Disease Prevention and Control reported that approximately 4 million patients per year are estimated to acquire an HAI in European hospitals. The number of deaths occurring as a consequence of these infections is estimated to be at least 37,000^1^. In US hospitals, it is estimated that each year there are 722,000 infections, with a 10% of mortality. Approximately 50,000 deaths per year are related to catheter infection. HAIs result in an estimated $30 billion in excess healthcare costs nationally each year^2^. Considering this economical impact it becomes clear that successful prevention and control strategies are highly cost-effective. HAIs prolong the suffering of the patients, increase health care costs, have other direct and indirect economic implications (loss of productivity and disability) and represent a reservoir for the emergence of additional, i.e. multiple antimicrobial resistance traits.

Modern healthcare employs many types of invasive devices and procedures to treat patients and to help them recover. Infections can be associated with the devices used in medical procedures, such as catheters or ventilators. HAIs include central line-associated bloodstream infections, catheter-associated urinary tract infections, and ventilator-associated pneumonia. Infections may also occur at surgery sites, known as surgical site infections^2^.

Biofilms play a pivotal role in healthcare-associated infections (HAIs). Typical antibiotic therapies are ineffective against biofilm formation and promote fast development of resistances^3^. New and novel approaches to prevent and treat biofilm infections are urgently required. Implant biofilm infections *Staphylococci*, in particular *Staphylococcus aureus* and *Staphylococcus epidermidis* are a leading cause of healthcare associated infections. Coagulase negative staphylococci are responsible for majority of the catheter related infections^4^.

In recent years, the interest towards natural and synthetic polymers has steadily grown owing to their physicochemical properties that can be easily modified to fulfil different tasks. Thermoplastic polyurethanes (TPU) represent an important class of thermoplastic elastomers with wide biomedical application as components of medical devices, scaffolds for tissue engineering and matrices for controlled drug release^5^. Another polymer widely used in biomedical applications is poly(dimethylsiloxane) (PDMS). It has been used in various medical devices, primarily because of their exceptional stability, good biocompatibility, low toxicity^6^.

Antimicrobial polymers can help to prevent the formation of biofilm-associated infections and to solve the problems associated with the use of conventional antimicrobial agents, such as residual toxicity, short-term antimicrobial activity and development of resistant microorganisms^7^. Antimicrobial polymer additives are often based on organic compounds or some metals (silver, copper, zinc). Organic antimicrobials often have thermal-decomposition temperatures similar to the temperature of the polymer-processing window; on the contrary, inorganic systems tend to be much more thermally stable. Some methods to introduce antimicrobial polymer modification are:^7–9^ modification of polymer surface properties without an antimicrobial agent, direct deposition of the antimicrobial agent on the polymer surface (silver coatings, tethered quaternary ammonium, synthetic antibiotics), chemical deposition of the antimicrobial agent of the polymer surface and direct incorporation of the antimicrobial agent in the polymer matrix (chlorhexidine, antibiotics). Each methodology has inherent advantages but also disadvantages such as low efficiency, technologically demanding, cost^8^.

On the other hand, glasses and ceramics are used extensively in thermoplastics and thermosets. Important functions for glasses and ceramics include enhanced processability and dimensional stability. A large variety of bioactive glass polymer composites has been investigated for bone tissue engineering^10^. Bioactive glasses have usually been combined with special antibacterial ions in order to achieve antibacterial properties. Most research in this field has dealt with the development of bactericidal Ag-doped bioactive glass polymer composites^11^.

To the best of our knowledge, there are no previous studies dealing with glass filled polymers, without any antimicrobial additives (e.g. silver, antibiotics), able to inhibit biofilms formed by bacterial pathogens typically associated with medical device-related infections. The goal of this study was to develop bactericidal glass filled polymer composites able to prevent biofilm formation, and to be used for biomedical applications. The glass selected as filler belongs to the B_2_O_3_-SiO_2_-Al_2_O_3_-NaO-ZnO system whose antimicrobial activity was tested in previous works^12^. This glass is non-toxic and shows excellent antimicrobial activity (logarithm of reduction > 3). This glass can be considered as a dispenser of Zn ions avoiding the health and environmental problems caused by dissolved Zn ions or nanoparticles. The presence of ZnO in the glass structure, acting either as a former or network modifier, controls the overall leaching behaviour, increasing its bactericidal life^12^.

The selection and evaluation of materials and devices intended for use in humans requires a structured program of assessment to establish biocompatibility and safety. Biocompatibility is the primary and paramount requirement for all medical devices. Hemocompatibility is relevant for all medical devices that come into large-area contact with the circulatory system. Therefore, additionally to the antimicrobial test, *in vitro* biocompatibility tests were carried out, ranging from the simple evaluation of the possible toxic characteristics of the developed composites (cytotoxicity assessment) to the complex type of compatibility (hemocompatibility).

## Experimental Section

### Materials

#### Polymers

Glass filled composites were formulated with two different commercially available polymers:

- One is a thermoplastic polyurethane (TPU): Pearlthane^®^ D16N85. It is a polyether-based TPU, supplied in pellet form for extrusion (Merquinsa, Barcelona).
- And the other one is a polydimethylsiloxane (PDMS): Sylgard^®^184. It was formulated according to manufacturer instructions (Dow Corning Corporation, Midland, MI). The weight ratio of base to curing agent was 10:1.

#### Biocidal glass

In previous studies the antimicrobial capability of glasses belonging to the B_2_O_3_-SiO_2_-Al_2_O_3_-Na_2_O-ZnO system, with increasing content of ZnO enriched glass, were probed against different microorganisms^12^. One of these glasses, labelled as ZnO35, with significant biocide activity against bacteria and yeast was selected as the bactericidal glass filler. The chemical composition of this glass was (in wt.%): 19.29 SiO_2_, 34.24 B_2_O_3_, 5.55 Na_2_O, 34.73 ZnO and 5.13 Al_2_O_3_. The glass was prepared by melting appropriate mixtures of reagent grade of SiO_2_, α-Al_2_O_3,_ B_2_O_3_, ZnO, and Na_2_CO_3_. The starting materials were mixed thoroughly, melted in platinum crucibles at 1250 °C, and then quenched by dipping into water. The glass powder was sieved to < 30 μm, and the corresponding average particle size was found to be d_50_ = 6.3 ± 0.1 μm. A full characterization of this glass was carried out previously by Esteban-Tejeda *et al*.^12^.

### Bactericidal composites

TPU composites were prepared by conventional melt processing method (extrusion compounding followed by compression molding). The antimicrobial glass was mixed with polyurethane, softened and processed by extrusion. Polyurethane were poured into the extruder from volume feeders in such proportions that composites of the glass contents equal to 5, 15, 25, 35, 50 wt% were obtained. A temperature of 175 °C, a rotor speed of 60 rpm and a mixing time of 4 min were employed. Thin films were prepared by compression moulding in a compression moulding press at 175 °C for 2 min. The prepared samples were labelled as P5, P15, P25, P35, P50, where numbers mean glass percentages. A reference sample made of polyurethane was also prepared and labelled as P. The series of composites and pristine polymers were refined by mechanical polishing using silicon carbide grinding discs (2400 grit), and then machined to obtain testing specimens of 0.6 cm diameter and approximately 0.1 cm thick.

PDMS composites were prepared by solution casting technique. The required loading of the filler was first mixed with silicone resin with constant stirring. After getting a homogeneous solution, stoichiometric amount of cure agent was added to the mixture and stirred for a further 5 min. The composite was immediately poured into rod moulds and left to cure under vacuum for two min and then under ambient conditions for 24 hours. The prepared samples were labelled as S5, S15, S25, S35, S50, where numbers mean glass percentages. A reference sample made of PDMS was also prepared and labelled as S. The series of composites and pristine polymers were refined by mechanical polishing using silicon carbide grinding discs (2400 grit), and then machined to obtain testing specimens of 0.6 cm diameter and approximately 0.3 cm thick.

### Characterization of the composites

Distribution of the dispersed glass particles into the polymer was evaluated by scanning electron microscopy (SEM) (Nova TM NanoSEM-FEI Company) using a secondary and backscattered-electron detector (vCD), with an accelerating voltage of 25.00 kV.

The three-dimensional (3D) micro-roughness pattern was examined using a Spectral Confocal Laser Microscope Leica TCS-SP2-AOBS. Three measurements were randomly taken in each one of the three different samples of each group. Data analysis and filtering were performed with Leica Confocal Software (LCS). A Gaussian filter was used to eliminate waviness and tilt. The values of all individual profiles evenly distributed along the surface were averaged to calculate roughness parameters using the freeware Confocal Uniovi Image. The average roughness (R_a_) and skewness (R_sk_) were quantified to evaluate potential differences between series of composites. R_a_ measures the overall surface roughness and R_sk_ evaluates the asymmetry related to the mean, thus determining whether roughness is mainly composed by valleys or peaks. Negative values of R_sk_ are associated with a pattern of roughness mainly composed by valley-like structures whereas R_sk_ > 0 reveals a predominance of peaks in the surface.

The tensile tests were carried out by using an INSTRON 5966 dual column tabletop universal testing system according to the ISO 527-3:1995, at a crosshead speed of 1.0 mm min^−1^. Five parallels for each sample were tested and the average value was reported.

### Antimicrobial testing

#### Microorganisms

Eight bacterial strains, including Gram-positive (*Staphylococcus epidermidis* ATCC 35984, *Staphylococcus epidermidis* SE, *Staphylococcus aureus* ATCC 29213 and *Staphylococcus aureus* SA) and Gram-negative (*Escherichia coli* ATCC 25922, *Escherichia coli* EC, *Pseudomonas aeruginosa* ATCC 23389 and *Pseudomonas aeruginosa* PA) organisms usually associated to biomaterials-related infections, were used to evaluate the antibacterial activity of the glass filled composites.

Reference strains (ATCC) were purchased from American Type Culture Collection. Clinical isolates (SE, SA, EC and PA) were obtained from intravascular catheters of patients with catheter-related infections at Hospital Clínico Universitario San Carlos, (Madrid, Spain). All strains were strong biofilm producers as determined spectrophotometrically by crystal violet staining method^13^ on polystyrene flat-bottom 96-well microtiter plates (Greiner bio-one, Kremsmünster, Austria) (data not shown).

Three to four colonies from a fresh culture on Columbia agar (BD Difco, Becton, Dickinson and Company, Franklin Lakes, MD), were growth overnight in an orbital shaker (100 r.p.m.) at 37 °C in Triptic Soy broth (TSB, BD Difco) supplemented with 50 mM glucose (Sigma-Aldrich, St Louis, MO; TSB-glu). Cells were harvested, washed twice in phosphate-buffered saline solution at 0.165 M and pH = 7 (PBS, Sigma-Aldrich), and adjusted to a 1 × 10^7^ cell ml^−1^ in TSB-glu.

#### Biofilm formation and quantification

Prior to biofilm production, TPU and PDMS discs were chemically sterilized, incubated in human serum for 12 h and washed twice with TSB-glu. Then, discs were placed in 96-well culture plates (Greiner bio-one) and 200 μl of each bacterial suspension (1 × 10^7^ cell ml^−1^) was added for 120 min at 37 °C. After the adhesion period, discs were washed with 5 ml of PBS, to remove non-adhered cells, placed into new wells of culture plates and incubated for 2 or 5 days in 300 μl of fresh TSB-glu medium.

After biofilm formation, discs were carefully washed three times with PBS, and singly placed in tubes with 0.3 ml of PBS. Tubes were vortexed for 1 min, followed by sonication at 37 KHz (Fisherbrand 15050, Thermo Fisher Scientific, Waltham, MA) for 10 min and by 1 min of vortexing to disperse bacterial cells. Suspensions were adequately diluted in PBS and plated onto Columbia sheep blood agar using a spiral platter workstation (Don Whitley Scientific, Shipley, UK). The plates were incubated for 48 h at 37 °C prior to colony forming units (CFU) counting. All experiments were performed in at least three times. Preliminary experiments indicated that sonication was not associated with a loss of viability of suspension cells.

#### Data analysis

Antibacterial effectiveness was expressed by the reduction of initial biofilm cell burden, %RBB, as the change (in percentage) of the number of viable cells within biofilm formed on glass filled polymer composites with respect the number of viable cells within biofilm in untreated polymers. The %RBB was calculated by the expression:

1 $$ \% RBB=\frac{100-(100\times In)}{It}$$

where *In* is the bacterial count (CFU ml^−1^) of the glass filled polymer composites, and *It* is the bacterial count (CFU ml^−1^) of the untreated polymers.

Comparisons of cell densities between untreated polymers and glass filled composites, and between maturation stages were performed using the two-tail t-test. Differences in the effectiveness of composites according to glass content were explored using analysis of variance (ANOVA) with the Tukey’s test for multiple comparisons. A P-value    < 0.01 value was considered significant.

#### Scanning electron microscopy (SEM)

Pre-formed biofilms on untreated and composites TPU and PDMS discs were fixed with a solution of PBS containing glutaraldehyde at 2.5% (Thermo Fisher Scientific) and paraformaldehyde at 4% (Thermo Fisher Scientific). After 1 h of incubation at room temperature, samples were dehydrated with a graded series of ethanol washes, followed by immersion in hexamethyldisilizane. After this, samples were dried with CO_2_ in a critical point dryer (Balzers CPD 030, Schalksmühle, Germany). The specimens were coated with gold/palladium and visualized in a scanning electron microscope (JSM-6400, JEOL JSM 6400; Jeol, Tohyo, Japan).

#### Zinc release

The series of composites and pristine polymers were immersed in 1 ml of Luria Bertani (LB) medium and kept under agitation during 2 or 5 days at 37 °C. The analysis of Zn^2+^ content released from the composites was analyzed by Inductively Coupled Plasma – Mass Spectrometer (Agilent 7700x ICP-MS).

### *In vitro* biocompatibility testing

Biocompatibility is one of the imperative and mandatory evaluations of materials for biomedical applications. *In vitro* biocompatibility evaluates cytotoxicity, heamocompatibility and blood clotting ability of the material, among others. Prior to biological assessment, all the specimens were cleaned by ultrasonication within ultrapure distilled water during 15 min (twice) and sterilized by a double cycle of ultrasonication within 70% v/v ethanol for 20 min.

#### Cytotoxicity

Cytotoxicity was tested, according to ISO 10993-5^14^, by measuring the mitochondrial activity of murine fibroblastic-like NIH-3T3 cells (ATCC) in culture using DMEM- Dulbecco’s Modified Eagle Medium supplemented with 5% new-born calf serum (NBCS), L-glutamine (1.5 mM) and penicillin/streptomycin (50 U ml^−1^ and 50 mg ml^−1^, respectively) at 37 °C in a humidified incubator at 5% CO_2_ (all reagents were obtained from Invitrogen). Three samples of each investigated material were incubated for 24 h at 37 °C in order to obtain complete extracts. These eluents were used as cell culture medium for fibroblasts (10^4^ cell ml^−1^) grown for 24 h (in triplicate). Cell viability was evaluated 24 h later using the colorimetric MTS assay (CellTiter 96 AQueous Non-Radioactive Cell Proliferation Assay; Promega Biotech Iberica, Spain), in which the MTS/PE reagent diluted in medium without phenol was added to cells for 2 h. Absorbance readouts at 440 nm were obtained by a BIORAD microplate reader.

#### Haemocompatibility

Haemolytic activity was evaluated by Drabkin’s method for haemoglobin estimation^15,16^. An elevated plasma haemoglobin level reflects erythrocyte membrane fragility in contact with materials. Pooled normal human whole blood anti-coagulated with Li-heparin was obtained from healthy donors (n = 3) under informed consent. Triplicates of each composite (6 mm diameter) were incubated with blood (1 ml per sample) for 4 h in a water bath (37 °C), inverting (twice) at 30 min intervals. After incubation, 100 µl of each supernatant were placed into a 96-well plate, adding then 100 μl of Drabkin’s reagent (Cromatest, Linear Chemicals SL; 1134015). The reaction mixture was allowed to stand a minimum of 15 min. Blood in the absence of materials was used as negative control. Hemoglobin and derivatives are oxidized to methaemoglobin by ferricyanide in alkaline medium. Methaemoglobin reacts with cyanide to form stable cyanmethaemoglobin that is spectrophotometry detected at 540 nm using a BIORAD plate reader. The cyanmethaemoglobin concentration (mg ml^−1^) was calculated from a standard curve ranging from 0.02 to 1.20 mg ml^−1^ performed with hemoglobin standard (bovine origin; Cromatest, Linear Chemicals SL). Plasma free haemoglobin was also titrated to ensure non haemolysis during blood extraction. The hemolytic index (percent of hemolysis), HI, was calculated using Eq. 2:

2 $$HI=\frac{Hb\,released}{Hb\,present}\times 100$$

where *Hb released* is the concentration (mg ml^−1^) of hemoglobin in plasma after blood incubation with the tested materials, and *Hb present* is the total concentration (mg ml^−1^) of blood hemoglobin in pooled blood.

#### Blood plasma coagulation assays

The effect of the investigated composites on extrinsic and intrinsic pathways of blood coagulation was evaluated from the prothrombin time (PT) and the inactivated partial thromboplastin time (uPTT). Fresh human blood was collected in BD Vacutainer^®^ tubes with 0.105 M sodium citrate from healthy volunteers under informed consent. The platelet poor plasma (PPP) was obtained *via* centrifugation of whole blood at 1500 g for 15 min. For each assay, pre-warmed samples of each composite (n = 3) were placed into test tubes. The tests for negative control were performed in tubes without materials. The PT assay was performed as per manufacturer’s instructions (TriniCLOT PT HTF; Tcoag Stago Group); PPP (100 µl) was added to test and control tubes and incubated at 37 °C for 3 min. Pre-warmed TriniCLOT PT (200 µl) was added to each tube. The clotting time at which a fibrous substance appeared was recorded in seconds. For uPTT assay, PPP (100 µl) was incubated with samples for 3 min at 37 °C. Then, rabbit brain cephalin reagent (100 µl; Pel Freeze Biologicals) was added, and the mixture incubated for 5 min at 37 °C. Pre-warmed calcium chloride 0.025 M solution (100 µl) was added to the diverse reaction mixtures and the time required to clotting recorded in seconds.

#### Statistical analysis

Data are presented as the mean value with the corresponding error of three independent experiments, except where otherwise stated, to ensure consistency. All statistical analyses were performed by the unpaired Student’s t test. A P-value < 0.05 was considered statistically significant.

## Results

### Surface characterization

**Figure 1:**
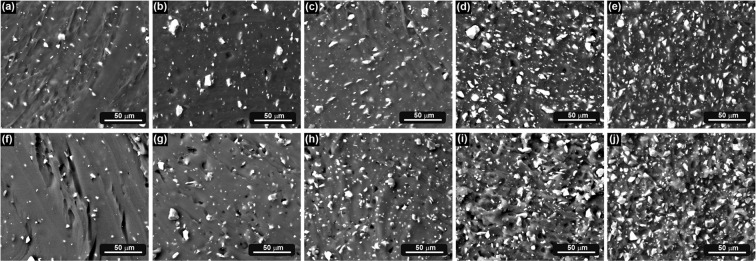
Scanning electron micrographs of TPU composites: (**a**) P5, (**b**) P15, (**c**) P25, (**d**) P35, (**e**) P50 and PDMS composites: (**f**) S5, (**g**) S15, (**h**) S25, (**i**) S35, (**j**) S50.

Scanning electron micrographs revealed the dispersion quality of the filler in the polymer matrices (Fig. 1). Angular glass particles are evenly dispersed in both matrices (i.e. TPU and PDMS), showing a narrow particle size distribution.

The topographical analysis through confocal microscopy revealed that all surfaces were flat and smooth, with an isotropic roughness pattern, i.e., with similar features in all directions. Table 1 summarizes mean and SEM values of roughness parameters obtained in the diverse glass-filled PDMS and glass-filled TPU compared to its respective polymeric counterpart, Sylgard^®^184 or Pearlthane^®^D16N85. In overall, all the investigated composites presented R_a_ values in the submicrometric level, below 250 nm. Each group presented negative R_sk_ average, disclosing a predominance of valleys in the roughness profile. A statistically significance was found when R_sk_ average in the glass filled composites is compared to the corresponding pristine material (100% polymer). Despite undergoing the same polishing methodology for smoothing samples, the dissimilar nature of pristine polymers and composites induced slightly differences in topography at micrometric level.

**Table 1:** Roughness parameters: data are mean ± SEM values (n = 9) of R_a_ and R_sk_ for the PDMS and TPU polymers filled with ZnO35 glass (0, 5, 15, 25, 35, 50 wt.%).

	TPU composites						PDMS composites					
	P	P5	P15	P25	P35	P50	S	S5	S15	S25	S35	S50
R_a_ [µm]	0.23^a^ ± 0.01	0.09 ± 0.01	0.12 ± 0.02	0.06 ± 0.01	0.05 ± 0.01	0.12 ± 0.01	0.05 ± 0.01^a^	0.21 ± 0.01	0.19 ± 0.03	0.22 ± 0.01	0.21 ± 0.02	0.22 ± 0.02
R_sk_ [µm]	−1.91 ± 0.67	−2.08 ± 0.97	−3.89 ± 1.70	−13.66 ± 3.40	−0.08 ± 0.04	−15.16 ± 9.00	−0.02^a^ ± 0.01	−12.85 ± 7.55	−14.83 ± 7.90	−14.31 ± 4.30	−10.52 ± 7.40	−7.06 ± 4.85

Statistically significance (p < 0.05) is indicated by^a^.

### Mechanical characterization

Static tensile loading test were performed on composites using an Instron 5966 material testing system. The effect of the glass on the tensile properties of the composites is summarized in Table 2. The tensile modulus of glass-filled PDMS composites increased from 1.94 to 16.16 MPa, and the tensile strength increased from 1.54 to 4.33 MPa (approximately a 281% increase over neat) when the glass content increased from 5 to 50 wt.%. Elongation at break decreases from 112% (neat polymer) to 39% [composite with the highest concentration of glass (i.e. 50 wt.%)].

**Table 2:** Mechanical properties of neat polymers and glass-filled composites.

	TPU composites						PDMS composites					
	P	P5	P15	P25	P35	P50	S	S5	S15	S25	S35	S50
Young’s Modulus [MPa]	14.59 ± 2.97	35.97 ± 6.14	24.13 ± 0.40	32.24 ± 2.19	65.56 ± 1.05	73.85 ± 4.12	1.94 ± 0.48	4.16 ± 0.98	3.79 ± 0.49	6.19 ± 1.12	7.88 ± 1.94	16.16 ± 5.06
Tensile strength [MPa]	13.50 ± 3.68	11.13 ± 6.87	18.98 ± 2.76	14.54 ± 2.74	11.89 ± 3.25	10.42 ± 0.40	1.54 ± 0.55	1.81 ± 0.64	1.24 ± 0.44	2.57 ± 0.14	2.95 ± 0.67	4.33 ± 1.74
Elongation at break [%]	342 ± 0.40	442 ± 0.73	581 ± 0.44	456 ± 0.10	360 ± 0.40	332 ± 0.19	112 ± 0.34	64 ± 0.05	46 ± 0.19	62 ± 0.02	47 ± 0.06	39 ± 0.10

In the case of glass-filled TPU composites, the tensile modulus of composites increased from 14.59 to 73.85 MPa, and the tensile strength increased from 13.50 to 18.98 MPa (approximately a 141% increase over neat) when the glass content increased from 5 to 15 wt%. At higher concentration of glass (i.e. 25, 35, 50 wt.%), the tensile strength started to decrease. Elongation at break increases from 342% (neat polymer) to 581% when the glass content increased from 0 to 15 wt%. At higher concentration of glass the elongation at break started to decrease to a value similar to the neat polymer.

### Biofilm inhibition

**Figure 2:**
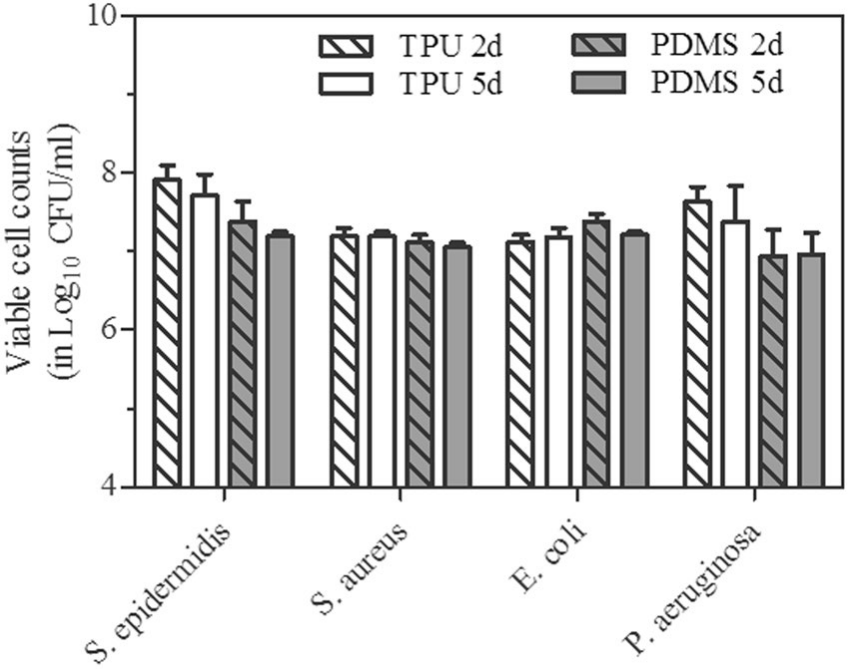
Evolution of viable cells recovered from biofilm formed on untreated TPU and PDMS polymers after 2 and 5 days of incubation. Results are the averages (±SD) of two strains (standard and clinical) per species.

Viable cells within biofilm recovered from untreated polymers on day 2 were in the range from 8.7 × 10^6^ to 7.9 × 10^7^, for all tested strains. The number of cells harvested from untreated discs on day 5 were not significantly different irrespective of the specie or strain tested (Fig. 2). There was no significant difference between cell burden found in biofilm formed on TPU or PDMS polymers, except for *S*. *epidermidis*, which formed significantly more dense biofilm on TPU (p < 0.05) irrespective of biofilm maturation stage.

Despite some intraspecies (ATCC vs. clinical srains) non-significant differences, the inclusion of glass on TPU or PDMS polymers significantly reduced biofilm cell densities in comparison with those in untreated polymers (Table 3). As shown in the table this effect, expressed by the %RBB, was species-dependent and increased in a glass content-dependent and in a time-dependent manner. The antibiofilm activity of composites increased as ZnO glass content increased against Gram-positive species and *E*. *coli* in TPU and PDMS polymers, and against *P*. *aeruginosa* in TPU. On day 2, the mean cells recovered from biofilms formed by *S*. *epidermidis*, *S*. *aureus*, *E*. *coli* and *P*. *aeruginosa* on TPU or PDMS composites was reduced between 64.1 to 98.5%, 20.6 to 79.6%, 39.1 to 66.9% and 46.8 to 80.6%, respectively, with respect cells number recovered from untreated polymers. Differences in the activity due to glass content (wt%) between TPU or PDMS composites were not statically significant.

**Table 3:** Antibiofilm effect of TPU and PDMS composites.

Species	Time (days)	Reduction in initial biofilm cell burden (in percentage, %RBB)^*^									
		TPU composites					PDMS composites				
		P5	P15	P25	P35	P50	S5	S15	S25	S35	S50
*S. epidermidis*	2	64.1 ± 25.1	64.4 ± 32.9	86.3 ± 14.7^a^	97.0 ± 1.3^a^	97.7 ± 2.0^a^	76.2 ± 25.8	83.3 ± 11.7	83.8 ± 17.5	92.4 ± 8.8^a^	98.5 ± 0.9^a^
	5	67.9 ± 10.3	87.8 ± 3.7^a^	98.7 ± 0.4^a,c^	99.1 ± 0.2^a,c^	98.9 ± 0.3^a,c^	89.5 ± 10.8	92.5 ± 0.4^a^	93.4 ± 3.6^a^	95.6 ± 0.9^a^	96.0 ± 4.3^a^
*S. aureus*	2	67.9 ± 16.5	72.9 ± 20.2	62.8 ± 32.9	72.4 ± 28.4	79.6 ± 1.3^a^	20.6 ± 11.7	20.4 ± 2.5	30.5 ± 6.6	22.5 ± 1.9	24.9 ± 6.7
	5	61.6 ± 5.2	86.0 ± 6.9^a^	92.4 ± 0.2^a^	95.6 ± 3.1^a,c^	97.9 ± 1.7^a,b,c^	30.1 ± 5.3	78.0 ± 4.4	85.0 ± 2.1^a^	90.2 ± 4.1^a,b^	94,3 ± 4.1^a,b^
*E. coli*	2	42.6 ± 11.6	60.2 ± 39.7	47.8 ± 19.6	39.1 ± 46.2	47.6 ± 26.7	46.2 ± 13.6	54.7 ± 2.4	60.8 ± 11.9	57.8 ± 16.5	66.9 ± 10.0
	5	84.4 ± 4.4^a^	88.5 ± 7.1^a^	90.9 ± 5.9^a,b^	87.0 ± 13.6^a,b^	95.2 ± 4.8^a,b^	88.3 ± 5.5^a^	92.6 ± 7.5^a,b^	89.9 ± 0.8^a,b^	95.1 ± 1.7^a,b^	92.0 ± 2.1^a,b^
*P. aeruginosa*	2	46.8 ± 19.6	45.7 ± 33.3	59.9 ± 37.0	79.6 ± 0.8^a^	80.6 ± 8.9^a^	—	—	—	—	—
	5	77.5 ± 26.0	76.1 ± 19.1	72.9 ± 17.8	83.7 ± 1.9^a^	93.8 ± 3.7^a^	34.7 ± 38.9^b^	23.9 ± 48.5^b^	—	—	—

^a^counts significantly lower than untreated polymers (*p* < *0*.*01*).

^b^increase in the antibiofilm activity on day 5 vs. day 2 (*p* < *0*.*01*).

^c^significantly more active than composites with 5 wt% of glass content.

^*^%RBB are calculated as the mean (±SD) reduction for each species (2 strains of *S*. *epidermidis*, 2 strains of *S*. *aureus*, 2 strains of *E*. *coli* and 2 strains of *P*. *aeruginosa*).

Prolonged incubation to 5 days has no significant impact in the effect of composites against *S*. *epidermidis* biofilms, regardless of ZnO content or the type of polymer used. However, the number of viable cells recovered in all composites was even lower than those obtained in day 2 (mean reductions between 67.9 to 98.9%). On day 5, viable counts were substantially reduced in biofilms formed on TPU and PDMS polymers by *S*. *aureus*, between 30.1 to 97.9% (p < 0.01 for P50, S35, and S50) and *E*. *coli*, 84.4 to 95.2% (p < 0.01 from P25 to P50 and from S15 to S50) and on TPU by *P*. *aeruginosa*, between 72.9 to 93.8%. Composites with 35 and 50 wt% glass contents were significantly more effective than composites with 5 wt% against Gram-positive species (Table 3). The effect of ZnO glass in biofilm formed by *P*. *aeruginosa* on PDMS substrates was still insignificant on day 5.

#### SEM analysis

**Figure 3:**
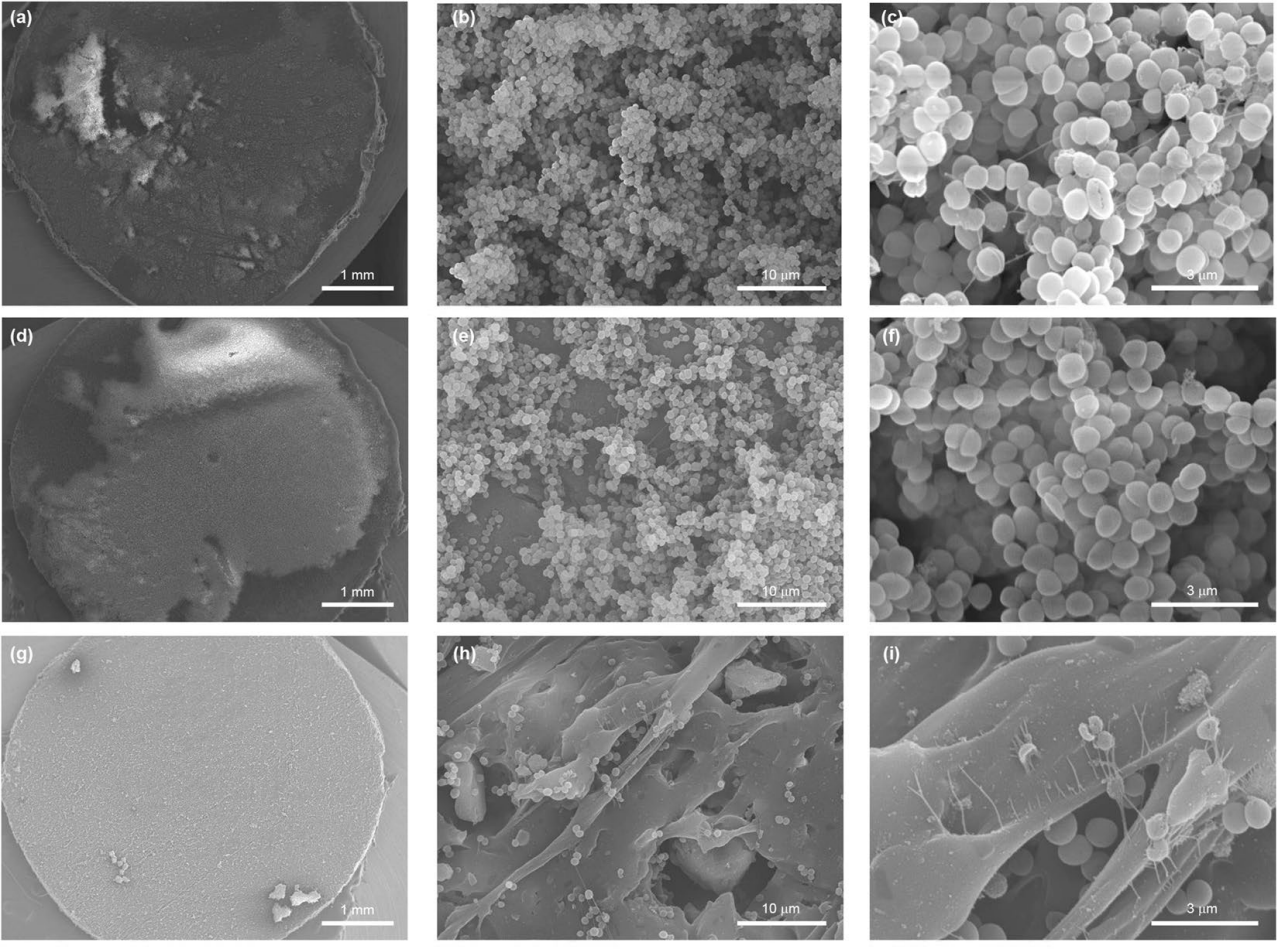
Scanning electron micrographs of *S*. *epidermidis* biofilm (5 days) formed on the surface of untreated TPU (**a–c**) and TPU composites with 5 wt% (**d–f**) and 50 wt% (**g–i**) of glass content. a, d and g panels, show the colonization of polymer surfaces by biofilms (magnification x20). The multilayer biofilm formed on untreated TPU is shown in the panel b (magnification x2.500) and c (magnification x10.000). Water channels and mucoid material appendages implicated in intercellular connection are observed. e and h panels (magnification x 2.500) and f and i panels (magnification x10.000) show the effect of the ZnO content in composites in biofilm architecture and cellular viability.

SEM analysis of untreated discs consistently revealed the same species-specific biofilm architecture regardless of the polymer used (data not shown). Images of biofilm formed by *S*. *epidermidis* on untreated and glass filled TPU composites after 5 days of incubation are showed in the Fig. 3. As shown in Fig. 3a, a thick three-dimensional biofilm structure was formed on untreated polymers. The biofilm was homogenous in distribution and abundant on the polymer surface. In glass-filled composites (Fig. 3d,g), biofilm were patchy in distribution and less abundant or even negligible with increasing glass content (Fig. 3g), which confirmed quantitative culture results. Biofilm seen in TPU composites with 5 wt% of ZnO glass (Fig. 3d–f), retain a tridimensional architecture but were thinner having fewer bacterial layers in comparison with those formed on untreated polymers (Fig. 3a–c). TPU composites with 50 wt% of ZnO glass (Fig. 3g–i) were found to be more clear with small cluster of cells scattered on the surface without forming biofilms. Signs of cell damage were apparent at this percentage of glass content (Fig. 3i). It is noteworthy that the roughness of the material favoured the colonization of polymer surface by bacterial cells. As shown in Fig. 3h,i, many cells cluster were found adhered within cavities generated on the polymer surface by the polishing. Similar results were found for PDMS composites (see Supplementary Fig. 1).

### Biocompatibility assessment

Biocompatibility depends on a number of factors such as physicochemical nature of materials, surface roughness, and wettability^17^. International standards determine the necessity of a complete biological evaluation of materials intended to fabricate medical devices^18^. Thus, the newly-developed glass-filled composites as biomedical devices have to be safe in terms of cytotoxicity, hemocompatibility and absence of interferences with coagulation mechanisms.

**Figure 4:**
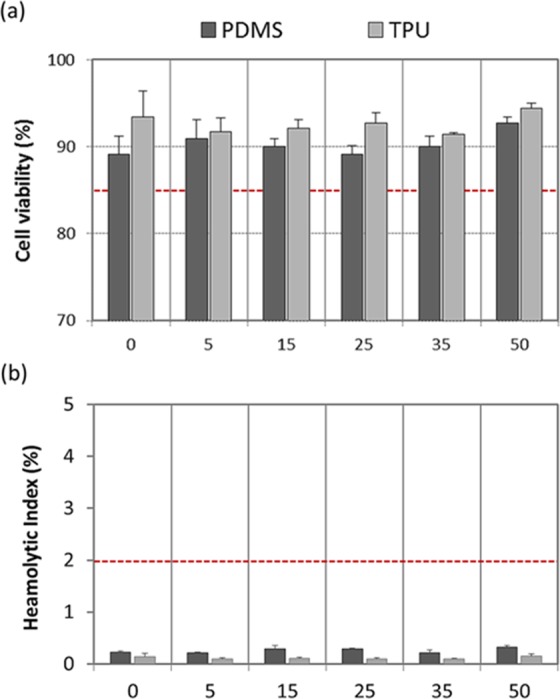
Biocompatibility: cellular compatibility (**a**) and haemocompatibility (**b**); cell viability rates higher than 85% rejected a potential cytotoxic effect and haemolityc index (HI) below 2% excluded haemolytic activity associated to glass-filled PDMS and glass-filled TPU systems with varying content of glass (0, 5, 15, 25, 35, 50 wt.%) (n = 6; error bars are SD). In each test, the red dashed line indicates the threshold.

Cytotoxicity can be considered as inversely proportional to cell viability. Cell viability was spectroscopically evaluated by quantifying the conversion of tetrazolium salts by mitochondrial dehydrogenases after incubation within extracts of PDMS and TPU composites including 0, 5, 15, 25, 35 y 50 wt.% of glass. Reduction of cell viability by more than 30% would be considered due to a cytotoxic effect^15^. Figure 4a shows cell viability rates obtained for the investigated materials; all of them were higher than 70%.

Haemolysis is a major problem associated with the destruction of red blood cells and the loss of cell function^19^. Figure 4b presents the quantified hemolytic index, HI, after exposure of whole blood to both the glass-filled composites and pristine polymers. ASTM F756-00 standard classifies biomaterials as non-haemolytic if values of HI are between 0–2%, as slightly haemolytic if values of HI are between 2–5%, and as haemolytic if values of HI are higher than 5%^16^. There was not significant release of hemoglobin after incubation with the glass-filled composites, and the haemolytic activity of all them was similar to that in the negative control, i.e. below 2%. Moreover, plasma free hemoglobin values below than 1 mg ml^−1^ (i.e. 0.35 ± 0.04 mg ml^−1^) ensured non haemolysis during phlebotomy. Total blood hemoglobin values of 120.23 ± 1.38 mg ml^−1^ encompassed within the physiological range (115–165 mg ml^−1^; as per manufacturer’s datasheet).

Hemostasis is a complex physiological process that prevents blood, including vasoconstriction, formation of a platelet plug and blood coagulation^20,21^. The inactivated partial thromboplastin time (UPTT) and prothrombin time (PT) tests were carried out to evaluate the coagulation activity of all the investigated materials. Fibrin polymerization started at 130 ± 5 seconds in the UPTT assay, and at 12 ± 3 seconds in the PT tests. In both tests, measured times in the presence of materials were not significantly diminished when compared with the negative control (blood without the sample). In addition, all these measured values in the PT and UPTT tests were encompassed within the expected values for adult healthy donors^22,23^.

## Discussion

It is well known that the degree of dispersion of particles in a polymer matrix is a governing parameter which controls the final properties of the resulting composites. In our case, SEM images of the composites (Fig. 1) prove the homogeneous distribution of glass filler in the polymer matrix. Taking into account the polarity of the polymers (hydrophobic) and the filler (hydrophilic), no interfacial adhesion will be expected.

As a result of conditioning procedures of substrates, slight differences in the roughness pattern are observed in composites compared to pristine polymers (Table 1); a higher predominance of valley-like structures in the glass-filled composites is revealed by more negative skewness values. This may be associated to pull-out in the second phase during mechanical polishing. Differences between PDMS and TPU systems can be attributed to hardness properties; Pearlthane^®^ D16N85 presents typical values of 85 Shore A whereas Sylgard^®^184 silicon has typical values of 43 Shore A (data from manufacturer’s datasheet). This would facilitate a more intense polishing on the PDMS system than on the TPU system. Nevertheless, considering the overall surface roughness, R_a_ values were less than 0.3 μm in all the investigated substrates (Table 1). Previous works have reported that significant differences in the adhesion of microorganisms are not observed on materials with R_a_ values of less than 0.9 μm^24,25^. Hence, the effect of the surface roughness of these glass-filled polymers is considered negligible in the experimentation.

The mechanical properties of glass-filled PDMS composites improved in terms of tensile strength and Young´s modulus, while elongation at break decreased with the addition of glass and as the glass content increased (Table 2). High efficiency reinforcement might be probably attributed to the uniform dispersion of glass in the polymeric matrix as well as to good glass-polymer interaction.

In the case of glass-filled TPU composites incorporation of 5–15% of glass particles provides positive effect on the tensile strength and elongation at break, as compared to neat polymer (Table 2). At higher concentration of glass, which is at 25, 35, 50%, the tensile strength started to decrease, which suggests that there was reduced quality of glass dispersion, presence of agglomerates or poor glass-polymer interactions, compared to the 5 and 15% counterparts. However, their mechanical properties can be considered quite similar to the pristine polymer.

The tensile properties of the obtained glass-filled composites are comparable with the literature data concerning biomedical polyurethane and silicone materials^26,27^. Changes in the mechanical properties must be taken into account depending on their intended use. Mechanical properties could be easily tuneable in the processing stage, through changes in the proportions of co-monomers, curing temperature, glass particle shape and size. In any case, the ultimate biomedical end use dictates the performance characteristics that must be evaluated.

Bacterial infection and colonization leading to biofilm formation remain major complications in medical devices and is an important step in the pathogenesis of infection, which frequently causes septic complications^3^. In the present work, the feasibility to diminish biofilm formation on the glass-filled polymeric composites was pointed out. The bactericidal glassy filler was not only active against collection strains but also against clinical organisms obtained from biofilm colonized medical devices (Table 3). It is important to note that coagulase negative staphylococci, *S*. *epidermidis* in particular, followed by *S*. *aureus*, are the most frequently causes of catheter-related bloodstream infections (BSIs), accounting for 37% and 13% of BSIs respectively^4^. Enteric gram-negative bacilli, including *E*. *coli* and *Pseudomonas* species are also frequently reported causes of BSIs (12% and 5% respectively). These formulated composites hold great promise and could offer some advantages to prevent biofilm formation compared to conventional antimicrobial therapies, i.e., systemic or catheter lock therapies, and the less cost effective strategies based on the use of coated or impregnated catheters with certain antimicrobial or antiseptic agents^28,29^ since prevalence of nosocomial multidrug-resistant strains are increasing^28^.

**Figure 5:**
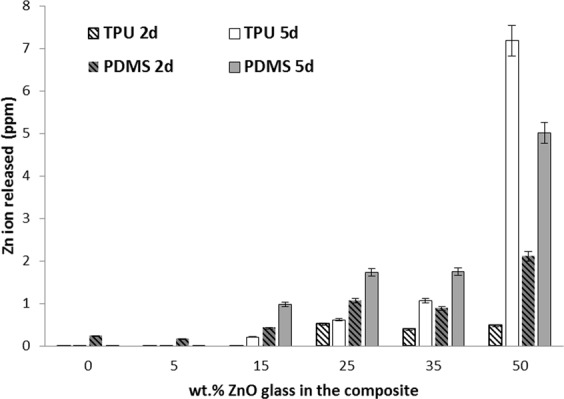
Zn ion release from glass-filled composites after 2 and 5 days.

The antimicrobial activity of this glass was previously probed against planktonic strains (*E*.*coli*, *S*.*aureus* and *C*.*krusei)*^12^. The obtained results from this previous study clearly indicated that Zn^2+^ released from the glass was responsible of the observed antimicrobial activity^12^. The operating mechanism in these composites is also attributed to the release of Zn ions from the glass particle surface. Zinc concentration was measured in the liquid media after two and five days (Fig. 5). The concentration of soluble zinc species in the broth is dependent of the concentration of the glass in the composites; the amount of Zn concentration leached out in the medium (i.e. LB) increased with increase in glass content in composite. Our results demonstrate that the antibiofilm effect of TPU and PDMS composites is related to the amount of zinc released from the polymer and therefore dependent on the length of exposure and the initial glass content preexisting in the polymer. The longer the exposure and the glass content, the greater the effect.

The antimicrobial properties of Zn^2+^ against planktonic strains and also against biofilms have been known since a long time^31,32^. The antimicrobial activities of soluble zinc species appeared strain-specific, dose- and time-dependent^30^. There are many suggested mechanism for the antimicrobial activity of Zn ions. Zn is an antibacterial ion which acts as an inhibitor of multiple activities in the bacterial cell, such as glycolysis, transmembrane proton translocation and acid tolerance^33,34^.

The similarities in the biofilm architecture found by SEM analysis (Fig. 3) between untreated polymers and composites with low glass contents, demonstrated that composites do not prevent the adhesion cell and biofilm formation on the polymer surface, at least at the challenged bacterial densities used which does not reflect the human intravascular catheter infection. This confirms a mechanism of activity based on the time-dependent release of an antibacterial compound.

On the other hand, Zn is an essential element for microorganisms and higher organisms because it is involved in many vital cellular reactions^35^. ZnO is recommended as GRAS (generally recognized as safe) by Food and Drug Administration of the United States (21CFR182.8991)^36^. Zn also aids in wound healing and enhancing immune responses. Despite these advantages, it can be toxic at high concentrations. Metabolic activity of cells in culture was not disrupted in the presence of extracts obtained from the glass-filled composites. In fact, the threshold established in the reference standard is far outweighed for each tested composition (Fig. 4a). A potential cytotoxic effect related to the newly-developed glass-filled polymer systems is therefore rejected. Moreover, the prevention of haemolysis due to direct contact between red blood cells with foreign materials, such as those used to fabricate catheters, is important and necessary; destruction of red blood cells *in vivo* can lead to anemia, jaundice and other pathological conditions^37,38^. All groups of glass-filled composites demonstrated a good haemocompatibility and did not show any significant toxicity when exposed directly to human blood *in vitro* (Fig. 4b).

Catheters are used in many medical procedures, being particularly common their insertion into blood vessels. The presence of indwelling catheters can pose risks of infection but also of blood clotting^39^. General screening tests were used to measure the integrity of the extrinsic and final common (PT test) as well as the intrinsic (uPTT test) pathways of the coagulation cascade^40,41^. Shortening of the PTT following contact with a material under standard conditions would indicates activation of the contact phase of blood coagulation. No significant activation of the coagulation pathways was found, as there was not considerably accelerated fibrin clot formation in the presence of the synthesized materials with respect to the negative control. Hence, the obtained results revealed that glass-filled PMDS and glass-filled TPU composites did not present prothrombotic activity or evidence of hypercoagulability.

## Conclusions

The results obtained in this investigation show the feasibility to develop bactericidal glass-filled thermoplastic polyurethane (TPU) or polydimethyl siloxane (PDMS) polymers, able to minimize bacterial adhesion (>90% reduction) and prevent biofilm growth of some the most frequent pathogens implicated in the etiology of biomaterials-associated infections. These new composites present non-cytotoxic activity, haemocompatibility and absence of interferences in the blood coagulation. Further investigation will be required to more precisely delineate and validate all the findings *in vivo*. This bactericidal glassy filler could be easily implemented at industrial scale, being prone to be manufactured by conventional processing techniques.

## Supplementary information

Supplementary Figure 1. Scanning electron micrographs of *S. epidermidis* biofilm (5 days) formed on the surface of untreated PDMS (a and b) and PDMS composites with 5 wt% (c and d) and 50 wt% (e and f).
