# Supplementary material for: Bactericidal ZnO glass-filled thermoplastic polyurethane and polydimethyl siloxane composites to inhibit biofilm-associated infections
Source: Sci Rep. 2019 Feb 26;9:2762. doi: 10.1038/s41598-019-39324-w (PMC6391378; doi:10.1038/s41598-019-39324-w)
Supplement: Supplementary file 1 — Supplementary Figure 1. Scanning electron micrographs of S. epidermidis biofilm (5 days) formed on the surface of untreated PDMS (a and b) and PDMS composites with 5 wt% (c and d) and 50 wt% (e and f). [file 41598_2019_39324_MOESM1_ESM.docx]

**Bactericidal ZnO glass-filled thermoplastic polyurethane and polydimethyl siloxane composites to inhibit biofilm-associated infections**

Belén Cabal*^1,2*^*, David Sevillano*^3^,* Elisa Fernández-García^1^, Luis Alou*^3^*, Marta Suárez^1,2^, Natalia González*^3^*, José S. Moya*^1^_,_* Ramón Torrecillas*^1^*.

## ^1^Nanomaterials and Nanotechnology Research Center (CINN-CSIC) – Universidad de Oviedo (UO) – Principado de Asturias, Avda. de la Vega 4-6, 33940, El Entrego, Spain

*^2^Nanoker Research, Pol. Ind. Olloniego, Parcela 22A, Nave 5, 33660 Oviedo, Spain*

*^3^Microbiology Unit, Medicine Department, School of Medicine, Universidad Complutense, Avda. Complutense s/n, 28040 Madrid, Spain*

**Correspondence to: b.cabal@cinn.es*

**Supplementary information**

**Supplementary Figure Captions**

**Supplementary Figure 1.** Scanning electron micrographs of *S. epidermidis* biofilm (5 days) formed on the surface of untreated PDMS (a and b) and PDMS composites with 5wt% (c and d) and 50wt% (e and f) of glass content. a panel (magnification x1.500), c and d panels (magnification x2.500), b, d and f panels (magnification x5.000).

**
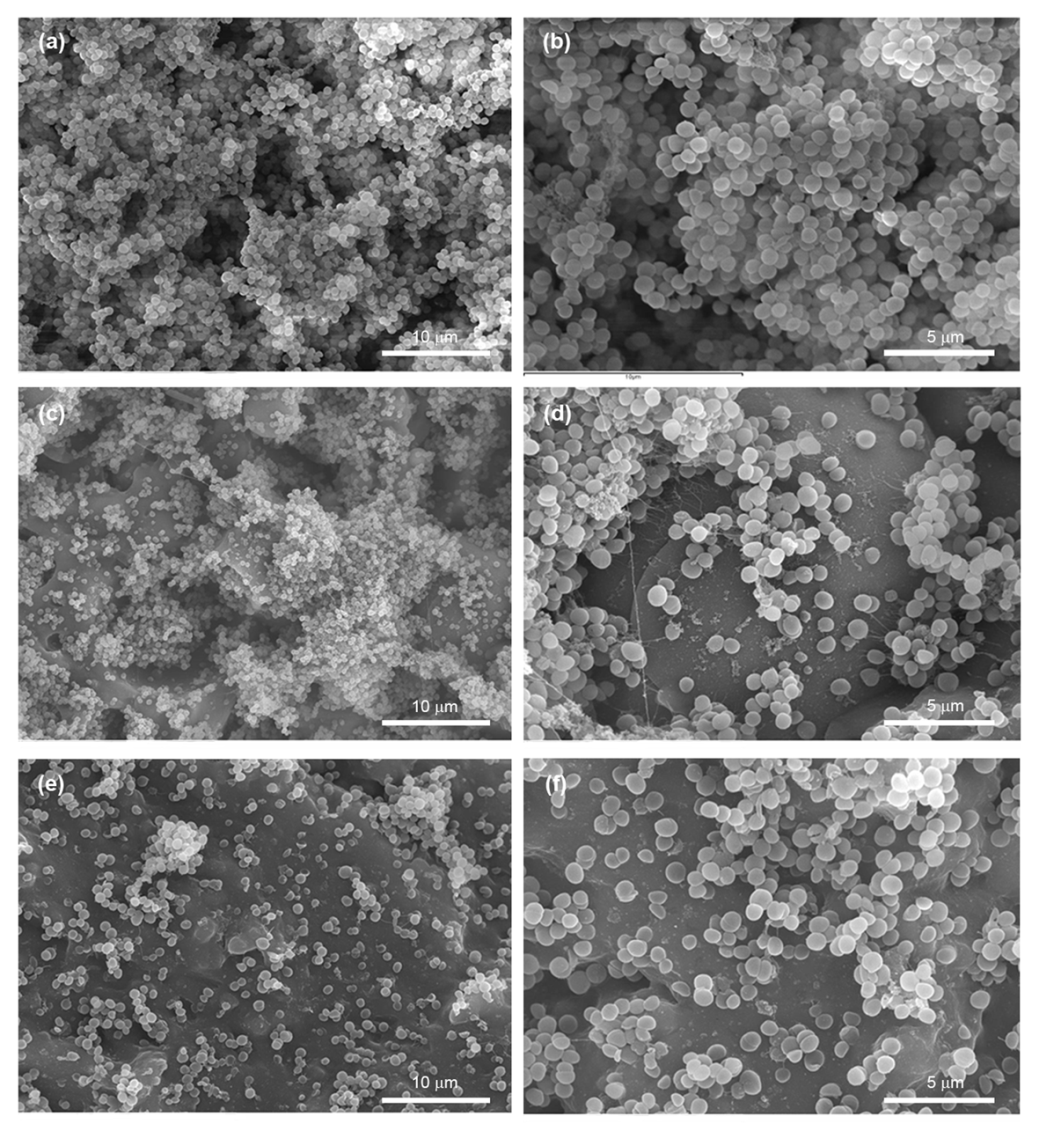
**

**Supplementary Figure 1.**
